# Emotion recognition training using composite faces generalises across identities but not all emotions

**DOI:** 10.1080/02699931.2016.1169999

**Published:** 2016-04-12

**Authors:** Michael N. Dalili, Lawrence Schofield-Toloza, Marcus R. Munafò, Ian S. Penton-Voak

**Affiliations:** ^a^ MRC Integrative Epidemiology Unit (IEU), University of Bristol, Bristol, UK; ^b^ School of Experimental Psychology, University of Bristol, Bristol, UK; ^c^ UK Centre for Tobacco Control Studies, University of Bristol, Bristol, UK

**Keywords:** Cognitive bias modification, emotion recognition, generalisation

## Abstract

Many cognitive bias modification (CBM) tasks use facial expressions of emotion as stimuli. Some tasks use unique facial stimuli, while others use composite stimuli, given evidence that emotion is encoded prototypically. However, CBM using composite stimuli may be identity- or emotion-specific, and may not generalise to other stimuli. We investigated the generalisability of effects using composite faces in two experiments. Healthy adults in each study were randomised to one of four training conditions: two stimulus-congruent conditions, where same faces were used during all phases of the task, and two stimulus-incongruent conditions, where faces of the opposite sex (Experiment 1) or faces depicting another emotion (Experiment 2) were used after the modification phase. Our results suggested that training effects generalised across identities. However, our results indicated only partial generalisation across emotions. These findings suggest effects obtained using composite stimuli may extend beyond the stimuli used in the task but remain emotion-specific.

Cognitive-bias modification (CBM) techniques are designed to modify the cognitive biases characteristic of a number of psychopathologies such as depression (Micco, Henin, & Hirshfeld-Becker, 2014) and social anxiety (Hoppitt et al., 2014). Since these biases are thought to be involved in the maintenance of these disorders, CBM techniques attempt to modify maladaptive cognitive biases, or induce adaptive biases. This allows the investigation of the causal relationship between these biases and an individual’s mood and behaviour (Hallion & Ruscio, 2011), and there is hope that these techniques may have therapeutic potential (Macleod, 2012).

A number of CBM techniques use stimuli depicting facial expressions of emotion, including attention bias (Britton et al., 2014; Shechner et al., 2014) and interpretation bias (Beard, Weisberg, & Amir, 2011) modification tasks. Two broad types of stimulus set are used: those where the image shown is a photograph of a single individual, such as the classic Ekman and Friesen (1976) faces or the NimStim face stimulus set (Tottenham et al., 2009), and those that use composite face stimuli (e.g. Penton-Voak, Bate, Lewis, & Munafò, 2012; Penton-Voak et al., 2013), where images of multiple individuals are averaged (Skinner & Benton, 2010). A potential advantage of using composite stimuli is that these eliminate idiosyncratic features, such as dimples or wrinkles, thus providing a representation of the emotion that contains the characteristics that are shared across individuals. Arguably, this offers a more generalised expression of the emotion, and potentially, stronger training effects. Furthermore, there is evidence that visual representations of facial expressions of emotion are encoded with reference to a prototype that can be approximated by an average of all emotional expressions (Skinner & Benton, 2010). Therefore, composite faces should present a more readily recognisable expression of a particular emotion, and one that perhaps generalises more widely than an individual exemplar emotional expression. However, images from these sets all depict the same composite face expressing different emotions. This makes it difficult to determine whether observed training effects on CBM tasks that employ computer-generated images are stimulus-dependent, as participants may be learning to respond to emotion expressions for only that particular face.

Emotion recognition training (ERT) is a CBM task used to shift the biased perception of neutral or ambiguous faces, characteristic of many disorders, from negative to more positive. ERT has shown promise in improving affect in individuals suffering from low mood (Penton-Voak et al., 2012), and reducing aggressive behaviour in youth at high risk of criminal offending (Penton-Voak et al., 2013). Other related techniques have increased perceptual sensitivity to facial expressions of emotion in male incarcerated violent offenders (Schonenberg et al., 2014), and improved emotional empathy and conduct problems in children with high levels of callous-unemotional traits (Dadds, Cauchi, Wimalaweera, Hawes, & Brennan, 2012). During the ERT task employed by Penton-Voak et al. (2012) and Penton-Voak et al. (2013), individuals identify images of facial expressions of emotion using composite faces from a continuum ranging from a negative emotion to happy. Using feedback, ERT shifts the balance point in the continuum, from where individuals are as likely to identify ambiguous faces as negative or positive, towards identifying more images as positive. However, existing CBM studies using ERT methods have only demonstrated training effects for the stimuli that the individual is trained on.

While ERT of this type appears to effectively shift biased perception of ambiguous faces from a negative emotion to a positive one, it is not clear whether this effect is emotion-specific (i.e. specific to the negative emotion individuals have been trained on), or extends to other negative emotions. If ERT generalises across emotions, trained individuals would perceive ambiguous faces as happy when these are presented as part of other happy – negative emotion continua as well. Alternatively, if ERT does not generalise across emotions, this suggests that the training is emotion-specific. It is also currently unclear whether effects of training using composite stimuli generalise well to other stimulus sets or if these effects are limited to the single identity presented during training.

Here, we present two studies that investigated the generalisation of emotion training using composite stimuli. In Experiment 1 we investigated whether ERT effects generalise across different identities using different composite stimulus sets. We hypothesised that training effects would generalise to new stimuli (i.e. a different individual). In Experiment 2 we investigated whether ERT effects are specific to the continuum individuals are trained on, or whether these effects generalise to other emotions.

## Methods

*Participants.* We recruited adults from the staff and students at the University of Bristol and from the general population for each experiment. Participants were recruited via email lists and word of mouth. On completion of the study session, participants were reimbursed £5 for their time and expenses or earned participant pool credit. The study was approved by the Faculty of Science Research Ethics Committee at the University of Bristol.

## Experiment 1

*Materials.* “Happy” and “sad” composite images were generated for both male and female stimulus sets from 12 individual male and 12 individual female faces showing a posed happy facial expression, and the same individuals of each sex showing a posed sad expression. Original images were collected from photographing individuals (primarily students) in Bristol. The 12 original images of each sex were each delineated with 172 feature points to construct the stimulus images, which allows both shape and colour information to be averaged across the faces to generate “average” happy and sad expressions using established techniques (Tiddeman, Burt, & Perrett, 2001). These composite images were used as endpoints to create a linear morph sequence that consists of images that change incrementally from unambiguously “sad” to unambiguously “happy”, with emotionally ambiguous images in the middle. We then created a sequence with 15 equally spaced images for use as experimental stimuli for each sex, resulting in two stimulus sets. Image dimensions were 585 × 765, and screen resolution was set at 1024 by 768 on a 17″ monitor.

The Positive and Negative Affect Schedule (PANAS) (Watson, Clark, & Tellegen, 1988) was used to assess positive and negative affect before and after the training task. It comprises 20 items scored on a 5-point scale, where 10 items assess positive affect and the remaining 10 assess negative affect.

**Figure 1.**
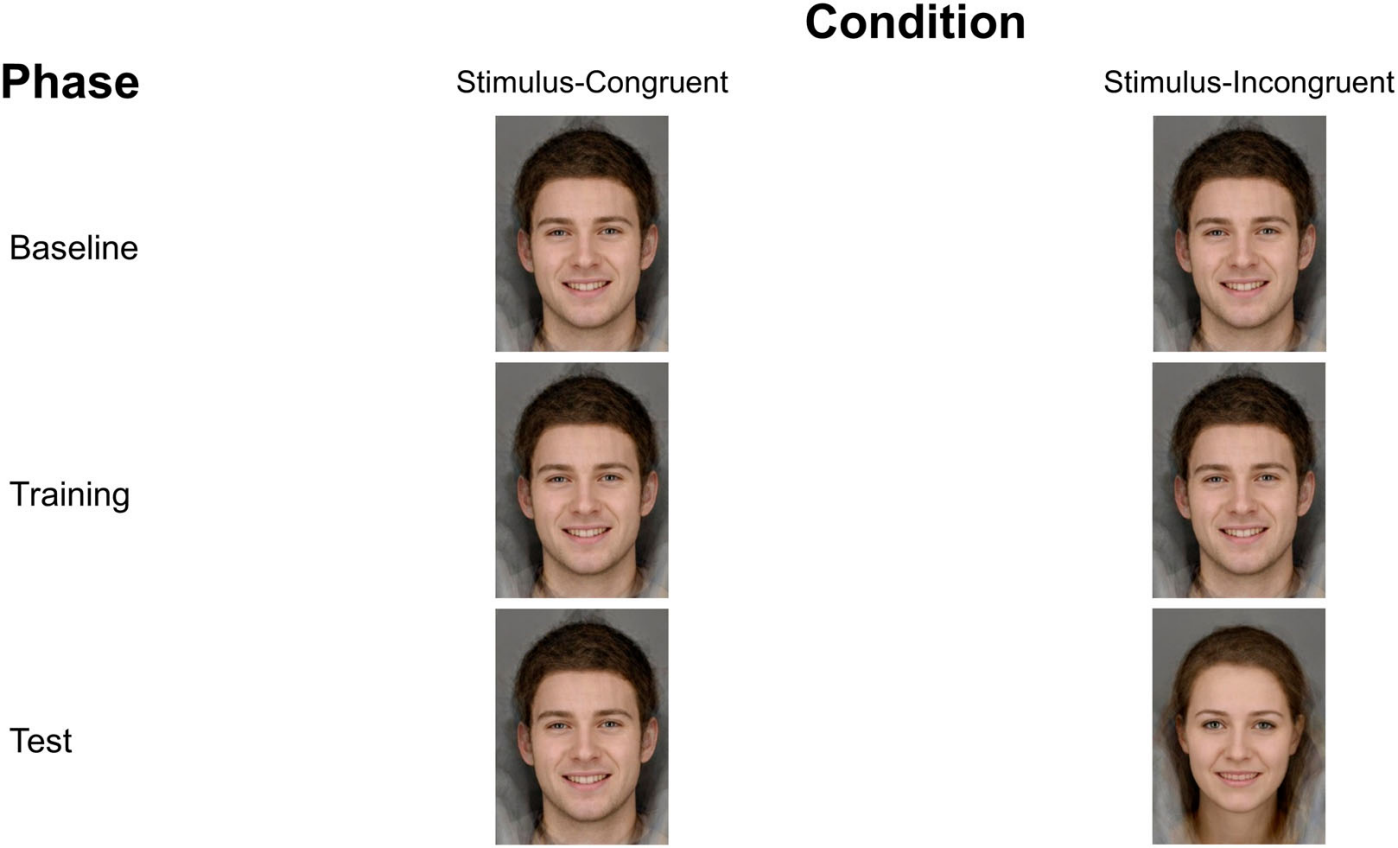
Example of stimulus set presentation for stimulus-congruent and stimulus-incongruent conditions by task phase.

*Procedure.* Once informed consent was obtained, participants were screened and randomised to one of four experimental conditions: two stimulus-congruent conditions, in which participants were trained and tested on the same stimulus set (either male or female), and two stimulus-incongruent conditions, in which participants were trained on one stimulus set (either male or female), and tested on a different stimulus set (either female or male). This is shown schematically in Figure 1.

Participants each completed the ERT task on a desktop computer while alone in a test room. The task consisted of three phases: a baseline phase, following by a training phase, and finally a test phase. The task’s baseline and test phases each consisted of 45 trials where each stimulus item from the morph sequence is presented three times. During the task participants were required to make a forced choice judgement as to whether the presented face was displaying a “happy” or “sad” expression. Each image was presented for 150 ms and was preceded by a fixation cross, presented for a random period ranging from 1500 to 2500 ms. To prevent processing of afterimages, following image presentation a backward mask of noise was presented for 250 ms, followed by a prompt that remained on screen until the participant made a key-press response (i.e. a judgement of “happy” or “sad”). Responses were made by pressing either the “c” key (labelled with a schematic image of a happy face) or “m” key (labelled with a schematic image of a sad face). After the baseline phase, each participant’s balance point (i.e. the point along the sequence where they were equally likely to judge the presented face as happy or sad) was estimated by calculating the number of “happy” responses as a proportion of the total number of trials. Trials in the training phase differed from trials in the baseline and test phases with the addition of feedback following participants’ responses. For all conditions, feedback was based on the participant’s baseline balance point, but the “correct” classification was shifted two morph images towards the “sad” end of the continuum. Therefore, the two images closest to the balance point that the participant would previously have judged as “sad” at baseline were considered “happy” when providing feedback. Feedback was a message saying “Correct!/Incorrect, that face was happy/sad”. As reported in an earlier study (Penton-Voak et al., 2012), the structure of the training blocks was designed to present more training trials depicting an ambiguous expression, than an unambiguous expression. Thus, images 1–2 (unambiguously happy) and 14–15 (unambiguously sad) were presented once in each training block, images 3–5 and 11–13 were presented twice in each training block, and images 6–10 were presented three times in each training block, totalling 31 trials per training block. Participants completed six blocks. During the test phase, participants in the stimulus-congruent conditions responded to stimuli from the same stimulus set, depicting the same individual as the stimuli they were trained on. Participants in the stimulus-incongruent conditions were tested on stimuli from another stimulus set, depicting an individual of the opposite sex. Participants completed the PANAS before and after completing the task.

*Statistical analysis.* The sample size for this study (160 participants, 40 in each condition) provided 80% power at an alpha level of 5% to detect an effect size of *d* = 0.65 for our between-group comparisons, and of *dz* = 0.45 for our within-groups comparisons. Data were analysed using a one-way between-subjects analysis of variance (ANOVA) and paired- and independent-samples *t*-tests to compare responding to the stimulus sets between phases and conditions. First, to establish that participants had similar balance points before training we compared mean balance points at baseline between all four conditions. Second, to confirm that training had occurred, we compared mean balance points at baseline and at test in the two stimulus-congruent conditions. Third, to confirm that generalisation had occurred, we compared the mean balance points of the conditions where participants responded to the same stimulus set at test, but were trained on the same (congruent) or a different (incongruent) stimulus set. Fourth, to further confirm that generalisation had occurred, we then compared mean balance points for each stimulus set at baseline to responding to the same set at test in an incongruent condition. Finally, we combined these results of our second and fourth analyses meta-analytically. Each analysis consisted of two independent tests, the results of which were then combined into a single summary effect size and confidence interval. We tested whether the effect size differed across the two analyses (congruent and incongruent) using an interaction test. We compared mean positive and negative affect scores at baseline between all four conditions to determine whether there were differences that may influence subsequent task performance. We also conducted paired-samples *t*-tests to investigate differences in affect scores following training across conditions.

The data underlying these analyses (doi:10.5523/bris.1uhuxhpgzmqu21gli0vqt7mbpz) are available from the University of Bristol Research Data Repository (http://data.bris.ac.uk/data/).

## Results

A total of 160 participants (81 female) aged 18–39 years (*M* = 22, SD = 4) were recruited. Participants were randomised to one of four conditions (*n* = 40 per condition), stratified by sex to ensure an equal sex distribution. One additional female participant was misrecruited due to a randomisation error. The characteristics of participants are shown in Table 1.

**Table 1.** Descriptive statistics and measures at baseline and test for Experiment 1.

Condition	Demographics		Phase					
			Baseline			Test		
	Age Mean (SD)	*N* (% Female)	Balance Point Mean (SD)	PANAS Positive Mean (SD)	PANAS Negative Mean (SD)	Balance Point Mean (SD)	PANAS Positive Mean (SD)	PANAS Negative Mean (SD)
Congruent male	22.43 (4.91)	40 (50%)	7.18 (1.43)	28.88 (6.13)	12.88 (3.31)	8.73 (2.06)	23.43 (7.75)	11.78 (2.44)
Congruent female	21.78 (4.32)	40 (53%)	7.40 (1.81)	28.90 (6.35)	12.13 (2.60)	8.95 (2.00)	25.30 (6.82)	11.55 (2.35)
Incongruent male	21.93 (4.36)	40 (50%)	7.40 (1.57)	26.63 (6.92)	12.38 (4.31)	8.78 (2.32)	22.73 (8.06)	12.18 (4.64)
Incongruent female	21.40 (3.26)	40 (50%)	7.45 (1.74)	28.13 (6.16)	13.20 (3.97)	8.50 (1.90)	24.53 (7.62)	12.15 (3.14)

**Figure 2.**
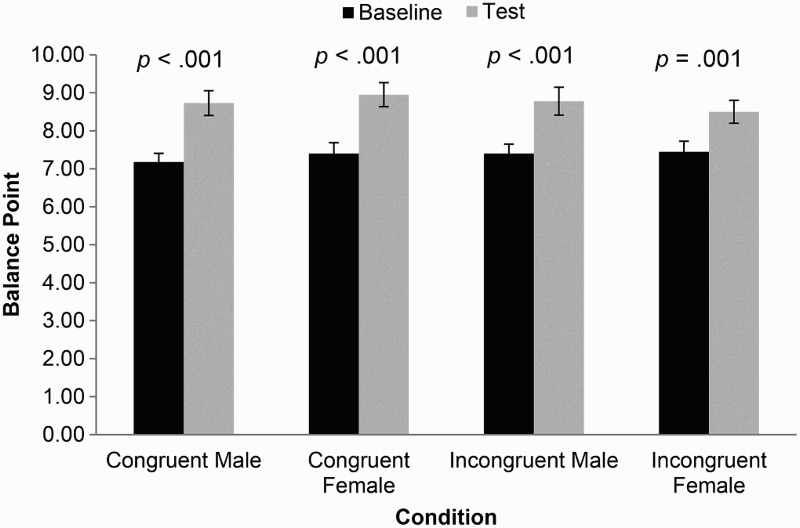
Balance point at baseline and test, by stimulus and condition for Experiment 1. Error bars represent standard error.

The mean thresholds at baseline were 7.2 (SD = 1.4) frames in the male stimulus-congruent condition, 7.4 (SD = 1.8) frames in the female stimulus-congruent condition, 7.4 (SD = 1.6) frames in the male stimulus-incongruent condition, and 7.5 (SD = 1.7) frames in the female stimulus-incongruent condition. The mean thresholds at test post-training were 8.7 (SD = 2.1) frames in the male stimulus-congruent condition, 9.0 (SD = 2.0) frames in the female stimulus-congruent condition, 8.8 (SD = 2.3) frames in the male stimulus-incongruent condition, and 8.5 (SD = 1.9) frames in the female stimulus-incongruent condition. These data are presented in Figure 2.

First, a one-way between-subjects ANOVA indicated no difference in threshold at baseline across all four conditions (*F* [3, 156] = 0.23, *p* = .879).

Second, paired-samples *t*-tests indicated clear training effects for both the male stimulus-congruent (mean difference −1.55, 95% CI −2.01 to −1.09, *p* < .001) and female stimulus-congruent conditions (mean difference −1.55, 95% CI −1.84 to −1.26, *p* < .001), with threshold shifted towards identifying ambiguous faces as happy rather than sad.

Third, and critically, independent-samples *t*-tests confirmed the transfer of training effects across identities, as participants responding to the same stimulus set at test responded similarly whether they were trained using that same set of faces (stimulus-congruent condition) or not (stimulus-incongruent condition) (mean differences −0.18 to 0.23, 95% CIs −1.14 to 1.11, *p*s > .61).

Fourth, to investigate whether training had an impact on how participants in stimulus-incongruent conditions responded to novel stimuli during the test phase, we investigated the difference between their responses at the test phase and responses to the same stimulus set at baseline from a stimulus-congruent condition. We assessed this for both stimulus sets using an independent-samples *t*-test and found evidence of a training effect for both the male (mean difference 1.33, 95% CIs 0.58 to 2.07 *p* = .001) and female (mean difference 1.38, 95% CIs 0.45 to 2.30 *p* = .004) sets. This indicates that participants in stimulus-incongruent conditions responded differently to stimuli viewed during the test phase when compared to participants in stimulus-congruent conditions responding to the same stimuli at the baseline phase, further confirming an effect of training.

Finally, combining the results of our second and fourth analyses meta-analytically, both stimulus-congruent (mean difference −1.55, 95% CI −1.79 to −1.31, *p* = 2.15 × 10^−37^) and stimulus-incongruent (mean difference −1.35, 95% CI −1.92 to −0.77, *p* = 4.01 × 10^−6^) conditions revealed strong evidence of a difference in responding to stimuli post-training. An interaction test found no difference between these effects (*p* = .520), providing additional support for the generalisation of task training effects from one identity to another.

A one-way between-subjects ANOVA indicated no clear difference between conditions at baseline for both positive (*F* [3, 156] = 1.11, *p* = .346) and negative (*F* [3, 156] = 0.72, *p* = .541) affect scores. Paired-samples *t*-tests indicated a decrease in both positive (mean difference 4.13, 95% CI 3.24 to 5.02, *p* < .001) and negative (mean difference 0.73, 95% CI 0.34 to 1.13, *p* < .001) affect scores on the PANAS following training across conditions. Positive and negative affect scores pre- and post-training by condition are shown in Table 1.

## Discussion

Our results indicate that ERT using composite faces generalises to other similar but non-trained faces. Since training effects have been shown to persist over several days (Penton-Voak et al., 2012), this suggests that the training effect may influence the interpretation of ambiguous emotional expressions in naturalistic settings. Given the decrease in both positive and negative affect post-task, we speculate that this flattening of affect may be associated with having completed the test session over approximately 20 minutes whilst alone in an artificially-lit laboratory test room.

Given the generalisability of training effects between identities, our findings suggest that the perception of novel faces in a natural setting might be influenced by ERT. Based on these results, we also expect training effects of other CBM tasks using composite faces to similarly generalise to non-trained identities. However in a natural environment individuals will encounter a range of facial expressions of emotions rather than just the two emotions they have been trained on. Therefore, in Experiment 2 we investigated whether training effects are specific to the trained emotions only, or whether these training effects generalise when presented with an untrained emotional expression.

## Experiment 2

## Methods

*Materials.* “Angry” composite images were generated for both male and female stimulus sets from the same 12 individual male and 12 individual female faces showing a posed angry expression. Images were created using the same method described in Experiment 1. These “angry” images and the “happy” images from Experiment 1 were used as endpoints to create linear morph sequences that consist of images that change incrementally from unambiguously “angry” to unambiguously “happy”, with emotionally ambiguous images in the middle. We then created a “happy-angry” sequence with 15 equally spaced images for use as experimental stimuli as well as the “happy–sad” sequence used in Experiment 1, resulting in two stimulus sets. Image dimensions and screen resolution were identical to Experiment 1.

As in Experiment 1, the PANAS (Watson et al., 1988) was used to assess positive and negative affect before and after the training task.

*Procedure.* The procedure for Experiment 2 was similar to that of Experiment 1. Following informed consent, participants were screened and randomised to one of four experimental conditions: two stimulus-congruent conditions, in which participants were trained and tested on the same emotional stimuli (either sad or angry), and two stimulus-incongruent conditions, in which participants were trained on one stimulus set (either sad or angry), and tested on a different stimulus set (either sad or angry).

Participants each completed the ERT task as they did in Experiment 1 while participants were now required to make a forced choice judgement as to whether the presented face was displaying a “happy” or “sad”, or “happy” or “angry” expression depending on the condition they were randomised to. Responses were made by pressing either the “c” key (for a “happy” response) or “m” key (for a “sad” or “angry” response depending on the condition they were randomised to). During the test phase, participants in the stimulus-congruent conditions responded to emotional stimuli depicting the same emotion as the stimuli they were trained on. Participants in the stimulus-incongruent conditions were tested on emotional stimuli from another stimulus set, depicting another emotional expression (sad or angry) than the set they were trained on. All other task parameters were identical to those described in Experiment 1. Similarly, participants completed the PANAS before and after completing the task.

*Statistical analysis.* The sample size for this study was calculated on the same basis as Experiment 1. Analyses for this experiment were similar to those used in Experiment 1. Briefly, data were analysed using a one-way between-subjects ANOVA and paired- and independent-samples *t*-tests to compare responding to the stimulus sets between phases and conditions. We also combined results of our analyses meta-analytically and tested whether the effect size differed across the two analyses (congruent and incongruent conditions) using an interaction test. We compared mean positive and negative affect scores at baseline between conditions and conducted paired-samples *t*-tests to investigate differences following training across conditions.

The data underlying these analyses (doi:10.5523/bris.1uhuxhpgzmqu21gli0vqt7mbpz) are available from the University of Bristol Research Data Repository (http://data.bris.ac.uk/data/).

## Results

A total of 160 participants (80 females) aged 18–40 years (*M* = 23, SD = 4) were recruited. Participants were randomised to one of four conditions (*n* = 40 per condition), stratified by sex to ensure an equal sex distribution. One male participant was excluded from the analysis due to antidepressant use, missed at screening. The characteristics of participants are shown in Table 2.

**Table 2.** Descriptive statistics and measures at baseline and test for Experiment 2.

Condition	Demographics		Phase					
			Baseline			Test		
	Age Mean (SD)	*N* (% Female)	Balance Point Mean (SD)	PANAS Positive Mean (SD)	PANAS Negative Mean (SD)	Balance Point Mean (SD)	PANAS Positive Mean (SD)	PANAS Negative Mean (SD)
Congruent sadness	21.00 (2.31)	40 (50%)	7.30 (1.49)	28.83 (6.03)	13.73 (4.68)	8.68 (1.86)	25.34 (8.20)	12.85 (3.48)
Congruent anger	23.28 (4.94)	40 (50%)	7.73 (1.66)	29.85 (6.23)	13.10 (2.74)	9.35 (1.58)	26.65 (7.12)	12.75 (2.81)
Incongruent sadness	22.13 (4.30)	40 (50%)	6.93 (1.61)	27.80 (7.22)	13.58 (3.70)	8.00 (1.78)	23.68 (8.87)	14.25 (4.47)
Incongruent anger	23.69 (4.09)	39 (51%)	8.10 (1.05)	28.51 (6.69)	13.10 (4.19)	8.05 (1.62)	25.74 (8.43)	12.62 (3.86)

**Figure 3.**
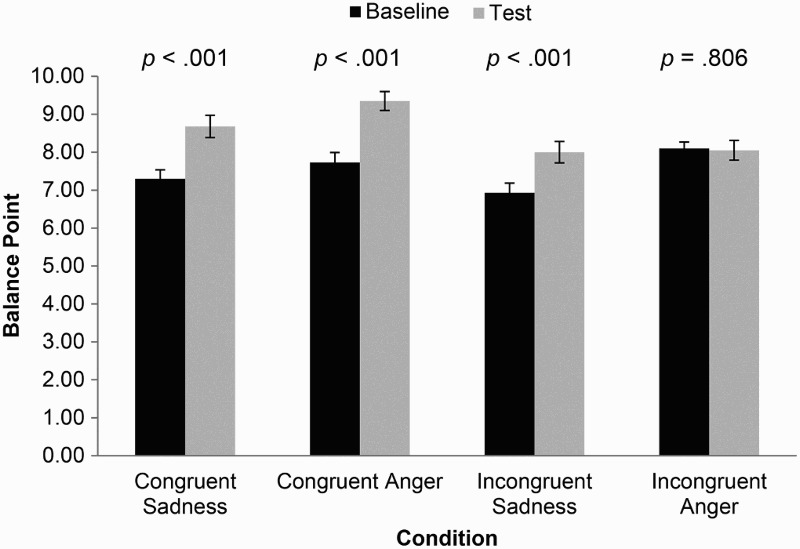
Balance point at baseline and test, by stimulus and condition for Experiment 2. Error bars represent standard error.

The mean thresholds at baseline were 7.3 (SD = 1.5) frames in the sadness stimulus-congruent condition, 7.7 (*SD* = 1.7) frames in the anger stimulus-congruent condition, 6.9 (SD = 1.6) frames in the sadness stimulus-incongruent condition, and 8.1 (SD = 1.1) frames in the anger stimulus-incongruent condition. The mean thresholds at test post-training were 8.7 (SD = 1.9) frames in the sadness stimulus-congruent condition, 9.4 (SD = 1.6) frames in the anger stimulus-congruent condition, 8.0 (SD = 1.8) frames in the sadness stimulus-incongruent condition, and 8.1 (SD = 1.6) frames in the anger stimulus-incongruent condition. These data are presented in Figure 3.

First, a one-way between-subjects ANOVA indicated a difference in threshold at baseline across all four conditions (*F* [3, 155] = 4.76, *p* = .003). A post-hoc Tukey test indicated that this difference was primarily between anger stimulus-incongruent and sadness stimulus-incongruent conditions (*p *= .003) with some evidence of a difference between sadness stimulus-incongruent and anger stimulus-congruent conditions (*p* = .076). We found no clear evidence of a difference in the remaining contrasts (*ps *> .57).

Second, paired-samples *t*-tests indicated strong training effects for both the sadness (mean difference −1.38, 95% CI −1.76 to −0.99, *p* < .001) and anger (mean difference −1.63, 95% CI −2.10 to −1.15, *p* < .001) stimulus-congruent conditions, with threshold shifted towards identifying ambiguous faces as happy rather than sad or angry.

Third, independent-samples *t*-tests suggested the transfer of training effects across emotions for sadness, as participants responding to the same stimulus set at test responded similarly whether they were trained using the same emotion (stimulus-congruent condition) or not (stimulus-incongruent condition) (mean difference 0.62, 95% CI −0.16 to 1.41, *p* = .116). However, this was not the case for anger, where participants in stimulus-congruent and stimulus-incongruent conditions responded differently at test (mean difference −1.35, 95% CI −2.10 to −0.61, *p* = .001).

Fourth, to investigate whether training had an impact on how participants in stimulus-incongruent conditions responded to novel stimuli during the test phase, we investigated the difference between their responses at the test phase and responses to the same stimulus set at baseline from a stimulus-congruent condition. We assessed this for both stimulus sets using an independent-samples *t*-test and found evidence of a training effect for sadness (mean difference 0.75, 95% CI 0.05 to 1.45, *p* = .035) but not for anger (mean difference 0.28, 95% CI −0.49 to 1.04, *p* = .48). This indicates that participants in sadness stimulus-incongruent conditions responded differently to stimuli viewed during the test phase when compared to participants in sadness stimulus-congruent conditions responding to the same stimuli at the baseline phase, further confirming an effect of training. However, this was not observed for anger conditions.

Finally, combining the results of our second and fourth analyses meta-analytically, the stimulus-congruent condition revealed strong support of a difference in responding to stimuli post-training (mean difference −1.48, 95% CI −1.77 to −1.19, *p* < .001), while the stimulus-incongruent condition offered weaker evidence of a difference (mean difference −0.54, 95% CI −1.04 to −0.03, *p* = .040). Consistent with the results of our earlier analyses, an interaction test indicated a difference between these effects (*p* = .002), suggesting partial generalisation had occurred.

A one-way between-subjects ANOVA indicated no clear difference between conditions at baseline for both positive (*F* [3, 155] = 0.67, *p* = .569) and negative (*F* [3, 155] = 0.27, *p* = .845) affect scores. Paired-samples *t*-tests indicated a decrease in positive affect scores (mean difference 3.41, 95% CI 2.67 to 4.16, *p *< .001) but no change in negative affect scores (mean difference 0.27, 95% CI −0.31 to 0.85, *p* = .36) on the PANAS following training across conditions. Average positive and negative affect scores pre- and post-training by condition are shown in Table 2.

## Discussion

Our results suggest that ERT using composite faces shows only partial generalisation to a novel emotion continuum: training effects for participants trained with sad faces generalised to angry faces, but training effects for participants trained with angry faces did not generalise to sad faces. This suggests that emotion-specific ERT would be most effective, and should target the emotion associated with an individual’s negative cognitive bias in order for training to occur. As in Experiment 1, our results again suggest that completing the training task appears to decrease self-reported positive affect, which we attribute to laboratory testing conditions, although we did not observe evidence for a change in negative affect.

Given our unexpected finding of partial generalisation, and based on our use of a limited number of emotions, we first recommend replication of this study before attempting to interpret or explain the mechanism underlying this result. While we cannot explain this finding, we can speculate as to why ERT might generalise from training on sad faces to angry faces but not from training on angry faces to sad faces. According to a classic dimensional model of emotion categories (Adolphs, 2002; Russell, 1980), both anger and sadness are classified as unpleasant or negative emotions. However, anger is classified as a high-arousal emotion (along with surprise and fear) while sadness is classified as a low-arousal emotion (along with neutral), with happiness and disgust classified as neither high- nor low-arousal. Based on our results, this might suggest that training on low-arousal emotions generalises to high-arousal emotions but that training on high-arousal emotions may not generalise to low-arousal emotions. We recommend that future studies investigating the generalisation of training effects between emotions assess generalisation between other negative emotions (e.g. fear, disgust) to determine whether this distinction between high- and low-arousal emotions may explain our partial generalisation finding.

As our results indicate that emotion recognition training effects do not generalise fully between emotions, this suggests that ERT does not simply induce a general positivity bias. This finding may extend to other CBM tasks, where training effects may also be specific to the particular emotion presented during the task. This stresses the importance of identifying the precise nature of the negative cognitive biases associated with mental illnesses in order to develop effective interventions, as these biases have been shown to vary with respect to the specific negative emotion associated with that particular illness (mood congruency effect) (e.g. Bower, 1981). Targeting the correct emotions associated with the specific negative cognitive bias (e.g. sadness in individuals suffering from low mood or depression) is crucial in modifying these biases.

## General discussion

Based on the results from these two experiments, the effects of ERT using composite faces generalise across identities but only partially generalise across emotions. Given these results we would expect a similar pattern of generalisation of training effects for other CBM techniques that also use composite faces. Therefore, we expect that the effects of CBM techniques using facial expressions of emotion stimuli for attention bias (Britton et al., 2014; Shechner et al., 2014) and interpretation bias (Beard et al., 2011) should similarly generalise to untrained faces. Similarly, our findings support the findings from past ERT trials (Dadds et al., 2012; Penton-Voak et al., 2012, 2013), suggesting these effects should extend beyond the laboratory. However, it is worth noting that the results of the current study demonstrate the generalisability of training effects between two composite stimulus sets. While promising, the findings from these experiments employ stimulus sets with limited variation in identity and emotion. Based on these experiments we can establish “close” generalisation of training effects, in a laboratory setting using composite stimuli, but additional testing is required to establish evidence of “distant” generalisation to novel faces in a naturalistic setting. In order to further investigate whether the effect observed in Experiment 1 would generalise outside of a laboratory setting, a comparison between a composite stimulus set and a classic stimulus set with images of single individuals would be necessary. This is especially of interest given the widespread use of classic stimulus sets in CBM tasks (Ekman & Friesen, 1976; Tottenham et al., 2009). However, while participants are seeing different emotions portrayed by the same “individual” in Experiment 2, we would expect this to facilitate rather than impair generalisation as the emotions are portrayed by a familiar, rather than novel, individual. Thus we would expect similar partial generalisation of effects across emotions in tasks using classic stimulus sets that present participants with images of multiple individuals.

While composite faces have potential practical and theoretical advantages, further work is necessary to establish their ecological validity relative to traditional face stimuli. However, our results suggest that ERT using these faces shows generalisability of training effects across identities but only partial generalisation among emotions, a finding that requires replication. Given that training effects appear specific to the emotions an individual is trained on, future research should investigate whether ERT is effective for individuals suffering from comorbid disorders, where negative cognitive biases affecting the perception of ambiguous faces may be associated with two or more facial expressions of emotion. This is an especially promising contribution to the CBM literature, suggesting that training effects across a number of tasks using emotional faces are likely to extend beyond the laboratory and are not identity-specific. However, it highlights the need to accurately identify and target specific emotions involved in the maintenance of mental illnesses in order for CBM to be most effective.
